# Hospital Progress in Paris.—II

**Published:** 1905-05-20

**Authors:** 


### HOSPITAL ADMINISTRATION.

### CONSTRUCTION AND ECONOMICS.

### HOSPITAL PROGRESS IN PARIS.—II.

By the Author of "Hospital Expenditure: The Commissariat."

(*Continued from page* 112).

The advantage of a decentralised hospital system are nowhere more forcibly illustrated than in the organisation of the staff. To look back over the history of ward management in English hospitals during the last thirty or forty years, is to survey a series of experiments carried out in different parts of the country, under widely varying conditions and not yet terminated. Let it be conceived for a moment that some central authority had dominated all the hospitals at the time when the inadequacy of the "old time" nurse began to be forced into prominence by surgical advance. Had this central authority been confronted with the problem, let us say in 1870, of laying down hard and fast regulations for the salary, diet, uniform, lodging, and instruction of all nurses and assistants in London hospitals, is it not evident that the institutions prepared to take the lead in reform must have been held back in conformity with the many for whom it was yet impracticable, and that the financial difficulties, hard in every individual case, must have proved insuperable when confronted simultaneously? To experiment when every step creates new problems and stirs ceaseless controversy over a widespread area is costly work, and when the money thus expended is public money, failure even in small matters becomes a dangerous weapon for the adversary. It is the special privilege of the voluntary hospital to attract support by virtue even of its experimental character and thus in perfect freedom to develop along natural lines.

The long delay in reforming the nursing administration in Paris hospitals has not been the result of any backwardness in perceiving the need for it on the part of the medical profession. It is due to the same causes which are at the present moment responsible for the procrastination of the Local Government Board in dealing with the status of the workhouse infirmary nurse. It is the difficulty of making comprehensive regulations to cover intricate and widely varying conditions, and the difficulty of finding money to place the service on a thoroughly satisfactory basis.

In 1880 the nursing department in all the Parisian hospitals was still under the control of the religious communities. Nuns supervised the work, and the services rendered to the patients were performed by men and women such as could be found to undertake very hard work at very small pay. The arrangement was found to be increasingly unsatisfactory, chiefly on account of the divided authority under which no doctor could be sure of getting his instructions carried out. Between 1880 and 1890 the laïcisation of the hospitals was gradually effected, and at present nuns are in charge only of the Hôtel Dieu and of the Boucicaut Hospitals, the latter being endowed under a recent will which provides for religious control.

Between 1874 and 1894 four schools for instructing the "infirmières" by whom the nuns and their lay assistants have been replaced were established at the following centres:—La Salpêtrière, Bicêtre, La Pitié, Lariboisière. Diplomas were granted to such pupils as were proficient, and in July, 1898, it was ascertained that this diploma had been gained by 1,963 out of the total number (3,318) of employés in the hospital. The standard is not a high one, and only a fifth of those holding the diploma could boast of even an elementary certificate of education.

The unsatisfactory conditions of diet, lodgings, pay, and hours which prevailed until recently in the Paris hospitals are responsible for the fact that a very large proportion of the infirmarians are men, and men of by no means a satisfactory type. "A workman without work, a servant without a place, a convalescent who has barely quitted the hospital, such is the man who from one day to another, provided his police record is good, is clad with the regulation uniform and dubbed 'infirmier.' The director who engages him has neither the time nor the means to choose. . . . This man knows nothing, and yet he must be trusted with the lives of 30 or 40 patients, perhaps more." Such is the complaint of the director-general of the service. Nor does it appear that the female infirmarian is of a higher order. The laïcisation of the hospitals appears in fact to have been a complete failure so far as any improvement in the ward administration is concerned, and the last few years have witnessed an incessant struggle on the part of directors and medical staff to train for the requirements of the service, persons incapable of instruction and unamenable to discipline.

The main lines on which the new reform is to be based are as follows:—

1. The male infirmarian is to disappear except for special cases.

2. A better class of female infirmarian is to be attracted to the service by means of higher pay, shorter hours of work, and improved quarters.

3. A second grade of hospital workers for general duties is to be formed entirely distinct from the infirmarian class, who will now be set free for attendance on the patients.

4. Probationers will be trained either for "hospice" or for hospital work. The period of training for service in the "hospice" is one year instead of the two which are compulsory on the hospital infirmarian. The distinction is reasonable since the French hospice is rather an almshouse than an institution for the reception of chronic patients.

The endeavour is to raise the position of the infirmarian till it approximates to that held by English and American trained nurses. The promoters of the scheme have spared no pains to discover the secret of the trained nurse's superiority and they are beginning to realise that it does not lie merely in facilities for hearing lectures, nor even in courses of practical instruction in nursing.

The part of the experiment which should be watched with most interest on this side of the water is the creation of a district branch of the hospital service for rough work outside the nurse's province. The existence of these workers is of course an integral part of the system of trained nursing. Yet the band of men and women servants, wardmaids, and scrubbers indispensable to the well being of the hospital is still unorganised in most English institutions, and the difficulty of finding suitable applicants is everywhere increasing. The proposal to enforce a six months' probation, and to divide the domestic staff into four classes, each promotion carrying an increase of privilege and pay from which not even the scrubber or charwoman is excluded, marks a praiseworthy attempt to attach a body of trained menial workers to the institution. In a floating population the regulation is excellent which provides that any worker removing from the neighbourhood may re-enter the service elsewhere in the same class as before.

To have skilled workers, experts in their own departments, if it be only in the polishing of a floor, right through an institution, is to get through the maximum of labour with the minimum of hands.

The French genius for organisation has been heavily handicapped in hospital management for many years by the weight of an inherently bad system imposed by a central administration. It has now an opportunity for proving its mettle, and, judging by progress achieved in countless other directions, it is not perhaps too bold to prophesy that in the course of the next ten years the Parisian-trained nurse will be fully abreast of her sisters in other nations, and that the Parisian hospitals will be second to none in *personnel*, organisation, and construction.

(*To be concluded.*)

